# Role of Anti-Inflammatory and Antioxidant Properties of Natural Products in Curing Cardiovascular Diseases

**DOI:** 10.3390/cimb47110955

**Published:** 2025-11-17

**Authors:** Amit Kulkarni, Chaitra Chidambar Kulkarni, Seetur Radhakrishna Pradeep, Jagadeesha Poyya, Avinash Kundadka Kudva, Vijay Radhakrishnan, Ajay Sathyanarayanrao Khandagale

**Affiliations:** 1 SDM Research Institute for Biomedical Sciences, Shri Dharmasthala Manjunatheshwara University, Sattur, Dharwad 580009, Karnataka, India; aggkulkarni4@gmail.com (A.K.);; 2 Department of Biotechnology & Crop Improvement, Kittur Rani Chennamma College of Horticulture (KRCCH), University of Horticultural Sciences, Arabhavi, Bagalkot 591218, Karnataka, India; 3 Division of Yoga & Life Sciences, Swami Vivekananda Yoga Anusandhana Samsthana (S-VYASA), Swami Vivekananda Rd., Jigani, Bingipura, Bangalore 560105, Karnataka, India; pradeep.sr23@gmail.com; 4 Department of Pediatrics, Penn State College of Medicine, Hershey, PA 17033, USA; 5 Department of Radiology, University of Missouri, Columbia, MO 65211, USA

**Keywords:** cardiovascular diseases, antioxidant, anti-inflammatory

## Abstract

Cardiovascular diseases (CVDs) remain a leading cause of mortality worldwide. According to the WHO, every year, there is an increase in the rate of death globally due to CVDs, stroke, and myocardial infarction. Several risk factors contribute to the development of CVDs, one of which is hypoxia, defined as a reduction in oxygen levels. This major stressor affects aerobic species and plays a crucial role in the development of cardiovascular disease. Research has uncovered the “hypoxia-inducible factors (HIFs) switch” and investigated the onset, progression, acute and chronic effects, and adaptations of hypoxia, particularly at high altitudes. The hypoxia signalling pathways are closely linked to natural rhythms such as the circadian rhythm and hibernation. In addition to genetic and evolutionary factors, epigenetics also plays an important role in postnatal cardiovascular responses to hypoxia. Oxidized LDL-C initiates atherosclerosis amidst oxidative stress, inflammation, endothelial dysfunction, and vascular remodelling in CVD pathogenesis. Anti-inflammatory and antioxidant biomarkers are needed to identify individuals at risk of cardiovascular events and enhance risk prediction. Among these, C-reactive protein (CRP) is a recognized marker of vascular inflammation in coronary arteries. Elevated pro-atherogenic oxidized LDL (oxLDL) expression serves as an antioxidant marker, predicting coronary heart disease in apparently healthy men. Natural antioxidants and anti-inflammatory molecules protect the heart by reducing oxidative stress, enhancing vasodilation, and improving endothelial function. For instance, the flavonoid quercetin exerts antioxidant and anti-inflammatory effects primarily by activating the Nrf2/HO-1 signaling pathway, thereby enhancing cellular antioxidant defense and reducing reactive oxygen species. Carotenoids, such as astaxanthin, exhibit potent antioxidant activity by scavenging free radicals and preserving mitochondrial integrity. The alkaloid berberine mediates cardiovascular benefits through activation of AMO-activated protein kinase (AMPK) and inhibition of nuclear factor kappa B [NF-kB] signalling, improving lipid metabolism and suppressing inflammatory cytokines. Emerging evidence highlights microRNAs (miRNAs) as potential regulators of oxidative stress via endothelial nitric oxide synthase (eNOS) and silent mating-type information regulation 2 homolog (SIRT1). While the exact mechanisms remain unclear, their benefits are likely to include antioxidant and anti-inflammatory effects, notably reducing the susceptibility of low-density lipoproteins to oxidation. Additionally, the interactions between organs under hypoxia signalling underscore the need for a comprehensive regulatory framework that can support the identification of therapeutic targets, advance clinical research, and enhance treatments, including FDA-approved drugs and those in clinical trials. Promising natural products, including polysaccharides, alkaloids, saponins, flavonoids, and peptides, as well as traditional Indian medicines, have demonstrated anti-hypoxic properties. Their mechanisms of action include increasing haemoglobin, glycogen, and ATP levels, reducing oxidative stress and lipid peroxidation, preserving mitochondrial function, and regulating genes related to apoptosis. These findings emphasise the importance of anti-hypoxia research for the development of effective therapies to combat this critical health problem. A recent approach to controlling CVDs involves the use of antioxidant and anti-inflammatory therapeutics through low-dose dietary supplementation. Despite their effectiveness at low doses, further research on ROS, antioxidants, and nutrition, supported by large multicentre trials, is needed to optimize this strategy.

## 1. Introduction

The global human population is nearly 8.2 billion, which raises significant concerns. In this rapidly growing population, mortality is a major issue. It is estimated that, in 2021, the most striking cause of nearly 20.5 million deaths was cardiovascular disease, according to a new report from the World Heart Federation (WHF). There are so many risk factors, and among these, chronic inflammation, hyperlipidemia, hypertension, obesity, sedentary lifestyle, overweight, diabetes mellitus, and genetic predisposition are contributing immensely [1,2,3].

Overproduction of reactive oxygen species (ROS) is one of the associated factors of these risk factors. ROS is a collective term that involves superoxide anions, hydroxyl radicals, nitric oxide, hydrogen peroxide, and peroxynitrite, among others [4,5]. When specific receptors of the innate immune system recognize microbes, they trigger inflammatory reactions to resolve infections and repair damage, thereby maintaining homeostasis equilibrium, a process known as the inflammatory response [6]. The formation and progression of atherosclerotic plaques are due to the increasing level of oxidative stress, and a lack of endogenous antioxidants is a significant cause of coronary heart disease [7,8].

Elimination of ROS and inflammatory agents in the body can be a promising approach for the treatment of cardiovascular diseases. An enzymatic and non-enzymatic antioxidant system is present in our biological system, which can scavenge and neutralize free radicals in a healthy person [9]. Excessive ROS production or insufficient scavenging mechanisms result in oxidative stress, which in turn may lead to the dysregulation of many biological processes involved in the pathogenesis of CVDs [4].

Glutathione peroxidase, catalase, and superoxide dismutase are among the endogenous antioxidant enzyme systems. Glutathione peroxidase reduces hydrogen peroxide and organic hydroperoxides into water and alcohols [10,11]. There is evidence that hypertensive humans or animal models under hypertension have shown less antioxidant defenses due to a decrease in enzymatic antioxidant activity [12,13,14,15,16,17].

Apart from our own biological system, many biomolecules have the capacity to act as potential antioxidants and anti-inflammatory agents, helping eradicate oxidative stress and inflammation in the body. The majority of such biomolecules originate in plants as a result of various metabolic processes. One such molecule is astaxanthin, a carotenoid that belongs to the xanthophyll group. Astaxanthin acts by donating electrons and binding with free radicals, thereby converting free radicals into non-reactive products. The study presented here involves both molecular and mechanistic aspects, with a major emphasis on clinical findings. The optimal doses of astaxanthin are 2–18 mg/day, with higher doses reaching up to 40 mg/day. This study encompassed both in vitro and in vivo strategies, including cell culture, mouse models, and human clinical trials [18].

Not only does astaxanthin exhibit antioxidant activity, but it also possesses anti-inflammatory properties. It was observed that 10μM supplementation of astaxanthin reported in a reduction in the expression of scavenger receptor class A and CD36 scavenger receptors in the Tohoku Hospital Pediatrics-1 (THP-1) macrophage line and reduced the expression of pro-inflammatory markers such as interleukin-1β (IL), IL-6, tumor necrosis factor-α (TNF-α), and cyclooxygenase-2 (COX-2). This study involved molecular-level investigations rather than clinical trials, with doses ranging from 1 to 10 uM, including an in vitro study on cellular models [19].

Hypoxia-induced oxidative stress can be mitigated using isoquercetin, a flavonoid isolated from the seed pods of *Cercis canadensis*. Reports say that isoquercetin inhibits ROS production in mitochondria, inhibits cytochrome C release, and induces apoptosis. This study is a molecular study rather than a clinical one. Isoquercetin was tested on H9c2 cardiomyocytes, a cellular model, at concentrations of 10, 20, and 40 μM [20].

Another example of flavonoid compounds found in citrus fruits is hesperidin, which possesses antioxidant properties. It regulates nuclear factor erythroid 2-related factor 2/antioxidant response element/heme oxygenase-1 (Nrf2/ARE/HO-1) and transforming growth factor beta/mothers against decapentaplegic homolog 2 (TGFβ1/smad3) signal transduction, which is a new target for the treatment of myocardial ischemia–reperfusion injury [21].

Both oxidative stress and inflammation are chronic conditions that can lead to the development of cardiovascular diseases, and they may also contribute to hypertension, type 2 diabetes, hypercholesterolemia, obesity, and even chronic kidney disease [22].

Cardiovascular diseases, including chronic heart failure, will occur because of inflammation response activation. This inflammation response will release inflammatory mediators, and these mediators have the potential to serve as relevant markers of disease severity. Markers such as *interleukins*, *cytokines*, *chemokines*, and *leukocyte adhesion molecules* serve as indicators of cardiac risk factors [23,24].

Hampering inflammation can be a promising approach to combat inflammatory responses. In this regard, medicinal plants are the best source of anti-inflammatory properties. Several medicinal plants have been reported to have anti-inflammatory activity. Many such plant-based biomolecules, which are termed secondary metabolites, have been identified and studied, and their potential effects have been revealed.

A few of them, which are uncommon anti-inflammatory molecules, are linolenic acid in *Ocimum sanctum*, releasing its anti-inflammatory action by blocking both cyclooxygenase and lipoxygenase, which are intimately involved in causing inflammation [25]. A triterpene dammarane-type saponin, called gensenosides, has been reported to exhibit anti-inflammatory properties, and it is a medicinal ingredient in Chinese herbal medicine [26].

The activation of Nrf2 plays a remarkable role in eradicating inflammatory responses. An alkaloid called Berberine (BBR), isolated from *Hydrastis canadensis*, exhibits this property, where, along with Nrf2 activation, it blocks mitogen-activated protein kinase (MAPK) pathways by inhibiting the nuclear factor kappa-light-chain-enhancer of activated B (NF-κB) signalling pathways and suppresses inflammation [27,28].

Hypoxia/reoxygenation (H/R)-induced reductions in ventricular myocyte survivability and tolerance to inflammatory damage are mediated by NF-B p65 subunit translocation, phosphorylation of inhibitor of kappa B (IB), and activation of inhibitor of kappa B kinase (IKK), effects that are influenced by ginkgolide C, a compound derived from *Ginkgo biloba* leaves [29,30].

Protection against CVD can be achieved by targeting the Nrf2 and NF-κB signalling pathways, and several natural compounds hold this potential. Ligustilide, a phthalide compound, inhibited vascular cell adhesion molecule-1 and intracellular cell adhesion molecule-1 (VCAM-1 and ICAM-1) and E-selectin expression by suppressing NF-*κ*B activation. It holds the capacity to induce Nrf2-mediated HO-1 expression [31].

Another pathway, TGF-β1-mediated SMAD pathway inhibition, can result in the attenuation of cardiac impairment following myocardial infarction (MI), and this can be achieved by Apigenin, which exhibits cytostatic and cytoprotective activity [32,33]. The nucleotide-binding oligomerization domain, leucine-rich repeat, and pyrin domain-containing 3 (NLRP3) inhibitors are also promising anti-inflammatory agents that can protect MI-induced myocardial fibrosis, and one such newly reported inhibitor is Oridonin isolated from *Rabdosia rubescens* [34].

**Figure 1:**
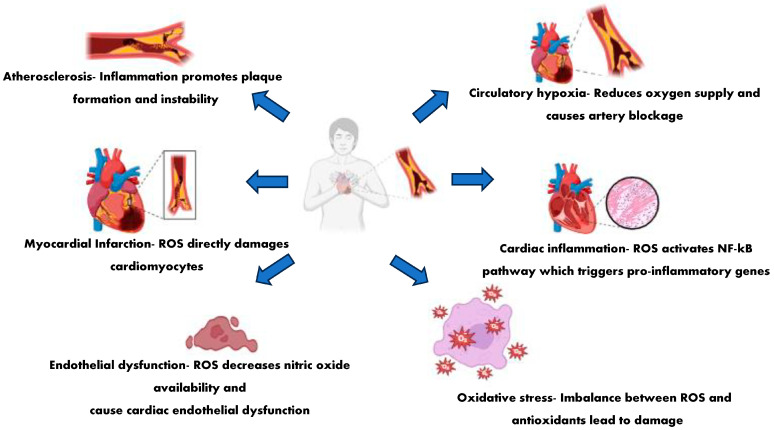
Different cardiovascular diseases occur due to oxidative and inflammatory stress.

Natural compounds with both antioxidant and anti-inflammatory effects, as a new approach to cardiovascular diseases, hold significant promise. Figure 1 shows the various cardiac abnormalities that result from oxidative and inflammatory stress. By targeting key pathological processes, such as oxidative stress, vascular inflammation, and endothelial dysfunction, these natural bioactive molecules can mitigate disease progression and improve cardiac outcomes.

The chemical architecture of natural compounds profoundly influences their biological activity, particularly in modulating oxidative stress and inflammation, which are key factors in cardiovascular diseases. Natural products encompass a wide variety of structural classes like polyphenols, alkaloids, terpenoids, flavonoids, glycosides, and others, each of which presents unique structural–activity relationships. In the case of polyphenols such as flavonoids, they possess multiple aromatic rings and hydroxyl groups, which hold the potential of antioxidant activity by donating hydrogen atoms to neutralize ROS, which is a prominent factor in inflammation and cardiovascular pathology. Even alkaloids with nitrogen-containing heterocyclic rings exhibit anti-inflammatory activity, mainly through the modulation of signaling enzymes and transcription factors involved in CVDs. Inhibition of pathways, such as inflammasomes like NLRP3 and kinases, is critically essential, facilitated by the presence of nitrogen, which allows for ionic and hydrogen bonding with enzyme active sites or receptor domains. The anti-inflammatory effects of terpenoids, including saponins and steroids, are also promising, as they have been shown to display anti-inflammatory properties by engaging steroid hormone receptors or modulating membrane fluidity and signaling [35]. The translation of antioxidants and anti-inflammatory molecules into clinical trials and treatments for human diseases can be a promising strategy, despite several significant challenges that are likely to arise. Poor solubility, stability, and absorption inefficiency when administered orally can limit their therapeutic potential. Being natural in nature, the safety and efficacy of the compounds pose another challenge that confronts approval difficulties from authorities, and it is also a time-consuming process [36].

In antioxidant and anti-inflammatory research, omics technologies are experiencing a paradigm shift, gaining in-depth knowledge of genes associated with oxidation and inflammation-suppressive properties. Genomic and transcriptomic sequencing approaches can now provide details about plant origins, biomolecules, and genetic and evolutionary backgrounds, which help modify and modulate them for future applications [36].

Beyond these positive values, clinical trials in humans, including those involving natural compounds, have yielded mixed results. Some interventional studies have shown little or no effects, and in some cases, adverse effects have also been reported [36]. The efficacy of therapy may depend on individual and specific compound interactions.

## 2. Hypoxic Conditions and Cardiovascular Diseases

Hypoxia, defined as an insufficient supply of oxygen to tissues, is a critical pathological condition that significantly contributes to the initiation and progression of CVDs. The heart, as a highly aerobic organ, is especially vulnerable to oxygen deprivation, which disrupts cardiomyocyte metabolism, impairs contractile function, and compromises overall cardiac homeostasis [37]. Hypoxic conditions are commonly associated with a spectrum of cardiovascular pathologies, including ischemic heart disease, MI, heart failure (HF), and obstructive sleep apnea, where they exacerbate myocardial injury and promote adverse cardiac remodeling [37,38].

A central mediator of the cellular response to hypoxia is *hypoxia-inducible factor-1* (*HIF-1α*). Under normoxic conditions, *HIF-1α* is rapidly degraded; however, hypoxia stabilizes this transcription factor, allowing it to translocate to the nucleus and regulate the expression of genes involved in angiogenesis, metabolism, erythropoiesis, and cell survival [37,39]. While *HIF-1α* activation facilitates short-term adaptation to hypoxic stress, its chronic upregulation contributes to maladaptive remodelling, including myocardial hypertrophy, interstitial fibrosis, and endothelial dysfunction, which collectively impair cardiac function [40,41].

Moreover, hypoxia plays a pivotal role in the pathophysiology of atherosclerosis, which remains a major underlying cause of ischemic heart disease. Within advanced atherosclerotic lesions, a hypoxic microenvironment develops due to increased metabolic demand from proliferating cells and inadequate oxygen diffusion caused by poor perfusion and vessel thickening [42]. Under these conditions, *HIF-1α* becomes stabilized, initiating the transcription of pro-angiogenic genes such as *VEGF*, which promotes intraplaque neovascularization [37]. However, the newly formed microvessels are typically fragile and leaky, allowing monocytes and other immune cells to infiltrate the plaque core [43]. These cells differentiate into macrophages and subsequently into foam cells, while activating inflammatory signaling pathways, particularly NF-κB, which further amplifies the local inflammatory milieu [44]. This chronic inflammation contributes to extracellular matrix (ECM) degradation, necrotic core expansion, and ultimately plaque instability, increasing the risk of plaque rupture and acute coronary syndromes, including myocardial infarction.

## 3. Role of Hypoxia and Hypoxia-Inducible Factors in Cardiovascular Disease

### 3.1. Hypoxia-Inducible Factor (HIF) Pathway

The cellular adaptation to hypoxia is primarily governed by HIFs, with *HIF-1α* acting as a central transcriptional regulator that becomes stabilized under low-oxygen conditions [45]. In normoxia, *HIF-1α* is hydroxylated by prolyl hydroxylase domain (PHD) enzymes, marking it for ubiquitination by the Von Hippel–Lindau (VHL) E3 ubiquitin ligase complex and subsequent proteasomal degradation [46]. However, under hypoxic conditions, this hydroxylation is inhibited due to reduced oxygen availability, allowing HIF-1α to accumulate, translocate into the nucleus, and dimerize with *HIF-1β* [47]. The resulting HIF complex binds to hypoxia-responsive elements (HREs) in the promoter regions of target genes, thereby regulating the expression of genes involved in angiogenesis, erythropoiesis, glucose metabolism, and cell survival—key processes essential for cellular adaptation to low-oxygen environments [48,49]. While acute *HIF-1α* activation initiates adaptive responses to re-establish oxygen homeostasis, chronic or excessive HIF signalling may contribute to maladaptive cardiac remodeling, fibrosis, and progressive functional decline [41].

Originally, HIF signaling was extensively studied in the context of tumorigenesis, where it promotes angiogenesis and metabolic reprogramming to support the survival of cancer cells under hypoxic conditions [43]. However, recent evidence suggests that *HIF-1α* plays non-pathological and reparative roles, including promoting cardiac angiogenesis, and is also involved in the pathophysiology of metabolic diseases, such as diabetes [49,50]. This dual role underscores the therapeutic potential of modulating the HIF pathway through various strategies, including gene therapy, stem cell therapy, protein delivery, and small-molecule chemical activators, which show promise for cardiac repair and tissue regeneration in preclinical and clinical models [47].

A family of iron critically governs the upstream regulation of *HIF-1α* stability and 2-oxoglutarate-dependent dioxygenases, known as PHDs. Among the three isoforms, *PHD1*, *PHD2*, and *PHD3*, *PHD2* (also known as *EGLN1*) is recognized as the primary oxygen sensor and the most crucial regulator of *HIF-1α* under both normoxic and hypoxic conditions [46]. Under normoxia, PHD2 hydroxylates conserved proline residues on the oxygen-dependent degradation domain of *HIF-1α,* promoting its recognition by the von VHL E3 ubiquitin ligase complex, thereby targeting it for proteasomal degradation [49,51,52]. However, during hypoxia, PHD2 enzymatic activity is significantly reduced due to limited oxygen availability, leading to HIF-1α stabilization, nuclear translocation, and subsequent activation of target genes involved in vasculogenesis, angiogenesis, re-epithelialization, and cell survival [37,53]. PHD knockout studies have shown that HIF activation can improve heart function following MI and ischemia–reperfusion (IR) injury. In particular, *PHD1* and *PHD3* knockout mice exhibited enhanced angiogenesis and improved cardiac function [54,55]. In recent studies, PHD2 inhibition showed preserved heart function, enhanced angiogenic factor expression, and decreased apoptotic markers after MI in Cardiomyocyte-Specific Prolyl-4-Hydroxylase 2 Inhibition on ischemic injury in a mouse MI model. Overall, cardiac *PHD2* gene inhibition is a promising candidate for managing cardiovascular diseases [56]. In another study, knocking out the cardiac-specific *PHD1* gene provides cardioprotection after myocardial infarction by activating key molecules, such as Insulin Receptor Substrate 2 (IRS2) and Heat Shock Protein Family A (Member 12B) (HSPA12B), involved in survival and angiogenesis. It also plays a novel role in cell differentiation and senescence, highlighting its therapeutic potential [57]. Overall, the HIF pathway represents a complex regulatory network balancing cellular adaptation and pathology in hypoxic cardiovascular tissues, making it a promising therapeutic target for the treatment of ischemic heart disease and heart failure.

### 3.2. Hypoxia-Induced Oxidative Stress and Inflammation in Cardiovascular Disease

Hypoxia significantly contributes to cardiovascular injury by inducing mitochondrial dysfunction, which leads to the excessive production of ROS, such as superoxide anions and hydrogen peroxide. These ROS cause oxidative damage to cardiomyocytes and vascular endothelial cells by attacking lipids, proteins, and DNA, thereby impairing cellular function and promoting apoptosis [58,59].

In addition to direct cellular injury, ROS act as signalling molecules that activate pro-inflammatory transcription factors, including NF-κB and activator protein 1 (AP-1) [60]. Activation of these pathways leads to the upregulation of pro-inflammatory cytokines, such as *IL-1β*, *IL-6*, and *TNF-α*, as well as vascular adhesion molecules, including VCAM-1 and ICAM-1. These factors facilitate leukocyte adhesion and infiltration into the vascular wall, sustaining chronic inflammation and exacerbating endothelial dysfunction [61].

Furthermore, hypoxia impairs the activity of eNOS, resulting in reduced production of nitric oxide (NO), a critical vasodilator and inhibitor of platelet aggregation. The consequent decrease in NO bioavailability promotes vasoconstriction, platelet activation, and a pro-thrombotic state, all of which increase the risk of acute cardiovascular events such as thrombosis and myocardial ischemia [62].

In recent years, studies have demonstrated that controlled hypoxic preconditioning, a brief nonlethal oxygen deprivation, triggers potent cardioprotective mechanisms by attenuating oxidative injury and inflammation. First, hypoxic preconditioning activates protein kinase C epsilon (PKCε) and mitochondrial ATP-sensitive potassium (mitoKATP) channels in cardiomyocytes, which significantly reduces the oxidative burst during reperfusion, thereby preserving cell viability and contractile function through early signaling rather than exogenous antioxidant delivery [62]. Mechanistically, the stimulation of mitoKATP channels opens inner mitochondrial pores, dampening ROS output at reoxygenation via a PKC-dependent cascade. Intermittent hypoxia-reoxygenation robustly activates transcription factors *HIF-1* and Nrf2, inducing antioxidant enzymes (such as superoxide dismutase, catalase, and glutathione peroxidase) and pro-survival targets such as vascular endothelial growth factor (*VEGF*), *erythropoietin*, and glycolytic genes, which bolster redox homeostasis and angiogenic capacity [63]. Disruption of Nrf2 abrogates this protective effect, underscoring its central role [64]. In one of the in vivo models, a study using intermittent hypobaric hypoxia shows improved left-ventricular function accompanied by decreased expression of oxidative markers (4-HNE, nitrotyrosine, etc.) and pro-inflammatory cytokines (*IL-1β* and *TNF-α*), despite no significant change in antioxidant enzyme levels, suggesting reduced ROS production rather than enhanced antioxidant capacity [65]. Collectively, these findings reveal a multifaceted protective network—spanning early mitochondrial signalling, transcriptional antioxidant programming, and reduced inflammatory mediator generation—highlighting the therapeutic potential of hypoxic conditioning in cardiovascular disease.

Natural compounds have emerged as key modulators of *HIF-1α* activity, enhancing its protective roles through multi-target interactions. This integration of HIF-1α biology with natural product pharmacology offers promising avenues for therapeutic modulation of oxidative and inflammatory diseases.

Squalene, naringenin, and apigenin, like plant-derived bioactive compounds, have shown potential in stabilizing and activating *HIF-1α.* These compounds may inhibit the default inhibitors of *HIF-1α*, such as *PHD2*, and prevent *HIF-1α* degradation via the pVHL pathway, leading to *HIF-1α* stabilization and enhanced transcriptional activity of antioxidant and anti-inflammatory genes [66].

Squalene positively modulates HIF-1α and downstream pathways, reducing inflammatory cytokines and oxidative stress markers. Botanical extracts, such as *L. aureum*, activate the HIF-1 signalling axis, thereby improving mitochondrial function and suppressing ROS and inflammation in cellular models [67].

### 3.3. Fibrosis and Cardiac Remodelling

Chronic hypoxia plays a pivotal role in driving pathological cardiac remodelling through the activation of profibrotic TGF-β/SMAD signalling. Under sustained low-oxygen conditions, TGF-β1 expression is elevated, leading to the phosphorylation and nuclear translocation of SMAD2 and SMAD3, which, in collaboration with SMAD4, upregulate genes encoding ECM proteins such as *collagen I*, *collagen III*, *fibronectin*, and connective tissue growth factor (CTGF) [68]. This cascade results in excessive myocardial fibrosis, stiffening the heart, reducing ventricular compliance, and impairing both systolic and diastolic function—a key contributor to heart failure progression. Moreover, hypoxia promotes cardiac fibroblast proliferation and their differentiation into myofibroblasts through caveolin-1/PTEN-mediated activation of PI3K/Akt, further amplifying ECM deposition [69]. Importantly, *HIF-1α* signalling emerges as a regulatory brake on excessive fibroblast proliferation and ROS accumulation post-injury; fibroblast-specific deletion of *HIF-1α* led to uncontrolled fibrosis and worsened cardiac dysfunction in mouse infarct models [70]. Together, these studies illustrate a complex interplay: while hypoxia-driven TGF-β/SMAD activation and PI3K/Akt–mediated myofibroblast conversion exacerbate fibrotic remodelling, *HIF-1α* exerts a counter-regulatory influence, limiting fibroblast-driven fibrosis by controlling mitochondrial ROS. These insights position both the TGF-β/SMAD and *HIF-1α* pathways as compelling therapeutic targets for mitigating hypoxia-induced cardiac fibrosis and preserving myocardial structure and function [71]. Likewise, the HP of cardiac progenitor cells (CPCs) upregulated *HIF-1α*, C-X-C motif chemokine receptor 4 (CXCR4), and anti-apoptotic genes such as *Bcl-2*, significantly enhancing cell survival, homing to ischemic myocardium, and fibrotic remodelling after myocardial infarction via the stromal cell-derived factor 1-alpha (SDF-1α/CXCR4) axis [72]. Furthermore, bone marrow mesenchymal stem cells (MSCs) subjected to hypoxia (1% O_2_) exhibited increased autophagy, which improved their survival post-transplantation and markedly reduced infarct size and fibrosis in murine MI models; however, these benefits were lost when autophagy was pharmacologically inhibited [73]. Additionally, in vitro studies demonstrate that HP of MSCs diminishes cardiac fibroblast activation and collagen deposition via a leptin-dependent suppression of the TGF-β/SMAD2 pathway [69]. Notably, emerging evidence also implicates extracellular vehicles (EVs) from hypoxic-induced pluripotent stem cells in delivering miR-302b-3p, which represses TGF-β/SMAD2 signaling and suppresses fibroblast-to-myofibroblast differentiation [74].

## 4. Anti-Hypoxic Properties of Natural Plant Compounds in Protecting Cardiovascular Complications

Cardiovascular diseases remain the leading cause of morbidity and mortality worldwide. A common pathological factor in many CVDs is hypoxia, which comprises insufficient oxygen supply to the myocardium and vascular tissues. Hypoxia triggers ROS generation, inflammation, endothelial dysfunction, and cell death, which exacerbate myocardial injury and vascular damage [69,73,74]. Managing hypoxia and its downstream effects is crucial to improving cardiovascular outcomes.

Recently, there has been growing interest in natural plant bioactive compounds for their potential to alleviate hypoxia-related damage in cardiovascular tissues. These compounds often act by scavenging ROS, modulating inflammation, enhancing mitochondrial function, and regulating HIFs, particularly *HIF-1α*, which orchestrates cellular responses to oxygen deprivation [75].

*HIF-1α* functions as a crucial transcription regulator at the interface of hypoxia, oxidative stress, and inflammation. Contemporary network pharmacology studies have employed bioinformatics, systems biology, and molecular docking to elucidate how bioactive compounds regulate antioxidant and anti-inflammatory responses, with a focus on *HIF-1α*.

For instance, squalene, a natural compound with documented anti-inflammatory properties, had been elucidated through a network pharmacology study. The analysis identified HIF1A as one of seven central targets mediating squalene’s effects. Regulatory enrichment identified PI3K-AKT, MAPK, and HIF-1 signaling pathways as the primary routes by which squalene mitigates inflammation and oxidative damage [76].

Another study used a systematic network pharmacology approach to investigate the antioxidant actions of *L*. *aureum*, a medicinal plant extract. The Kyoto Encyclopedia of Genes and Genomes (KEGG) pathway enrichment analysis identified the *HIF1α* signaling pathway as a dominant axis in antioxidative action. Medicinal plant potential enhanced HIF1α activity, which in turn improved mitochondrial function, suppressed ROS generation, and downregulated pro-inflammatory markers [77].

### 4.1. Flavonoids

Flavonoids are a diverse class of polyphenolic compounds abundantly found in fruits, vegetables, and medicinal plants. Their well-documented antioxidant, anti-inflammatory, and anti-apoptotic properties have been extensively studied for cardioprotective effects, particularly under conditions of hypoxia and ischemia [78].

Mechanism of Action:

They work by scavenging ROS and inhibiting lipid peroxidation, which are key drivers of cellular damage. They also activate antioxidant pathways, such as the Nrf2/HO1 pathway, and modulate pro-inflammatory cytokines, including TNF-α and IL-6.

Experimental Evidence:

Among them, quercetin, one of the most widely studied flavonoids, exhibits cardioprotective actions by scavenging ROS and inhibiting lipid peroxidation, key drivers of cellular damage during H/R injury. A study by Song et al. demonstrated that quercetin protects human coronary artery endothelial cells from hypoxia/reoxygenation-induced mitochondrial apoptosis by activating the Nrf2/HO-1 pathway, enhancing superoxide dismutase and catalase activity, restoring mitochondrial DNA copy number, and reducing ROS and malondialdehyde (MDA)—a protection reversed by Nrf2 knockdown [79]. Furthermore, another study showed that quercetin significantly improved cardiac function in rats with myocardial ischemia–reperfusion injury. Treatment with quercetin resulted in a significant reduction in pro-inflammatory cytokines (*TNF-α*, *IL-6*, and *IL-1*) and the cardiac injury marker CK-MB. These effects suggest a strong anti-inflammatory and cardioprotective role of quercetin, mediated in part through the activation of (mitoKATP) channels and the modulation of the NO signaling pathway [80]. Chang et al. reported that quercetin mitigates hypoxia/reoxygenation oxidative stress and ER stress in human cardiomyocytes via SIRT1/TMBIM6-dependent activation of mitophagy [81]. In a study on H/R cardiomyocytes and MI/RI rat models, quercetin activates the Sirt3/SOD2 pathway, reduces mitochondrial ROS, and enhances antioxidant defense, thereby protecting cardiomyocytes from ischemia–reperfusion injury and highlighting its potential as a mitochondria-targeted therapy for heart disease [82]. Lutein, a naturally occurring xanthophyll carotenoid found abundantly in green leafy vegetables, has emerged as a promising agent in protecting cardiovascular tissues against hypoxia-induced damage. A study in a rat model of myocardial I/R injury demonstrated that lutein administration significantly reduced myocardial infarct size, as evidenced by TTC staining. Additionally, lutein treatment also decreased serum levels of cardiac injury markers, including *CK-MB*, *lactate dehydrogenase (LDH)*, and *aspartate transaminase (AST)* [83]. Echocardiographic assessments showed that lutein treatment improved left ventricular ejection fraction (LVEF) and reduced left ventricular end-systolic and end-diastolic volumes (LVESV and LVEDV), indicating enhanced cardiac function [84].

Hesperidin, a flavanone glycoside predominantly found in citrus fruits, enhances NO bioavailability by upregulating eNOS activity, leading to improved endothelial-dependent vasodilation [85]. Researchers demonstrated that hesperidin significantly reduced arrhythmias, myocardial apoptosis, and infarct size in a rat model of I/R injury. Hesperidin treatment led to increased myocardial nitrite levels, enhanced antioxidant capacity, and reduced inflammation [86]. The experiment reported that 200 mg/kg of hesperidin pretreatment for 3 days markedly decreased infarct size, myocardial enzyme levels (CK, LDH), apoptosis, inflammation, and oxidative stress following I/R in rats. These cardioprotective effects were mediated through activation of the PI3K/Akt pathway and inhibition of the pro-inflammatory protein high mobility group box-1 (HMGB1) [87].

### 4.2. Alkaloids

Alkaloids, nitrogen-containing compounds with diverse biological activities, exhibit considerable potential in protecting cardiovascular tissues under hypoxic conditions [88].

Mechanism of Action:

Their mechanisms include inhibiting key pathways such as NF-kB and PI3k/Akt, modulating autophagy, and promoting mitochondrial biogenesis.

Experimental Evidence:

Berberine, an isoquinoline alkaloid extracted from plants such as *Coptis chinensis*, exhibits significant protective effects against hypoxia-driven cardiovascular injury. In the rat model of myocardial ischemia–reperfusion (I/R) injury, berberine reduced infarct size, lowered serum cardiac markers (*CK-MB*, *LDH*, and *troponin I*), and preserved cardiac output by attenuating mitochondrial dysfunction and apoptosis. This was evidenced by the increased *Bcl-2/Bax* ratio and decreased cytochrome c release in cardiac tissue [89]. In another study, berberine inhibited excessive autophagy in H/R models by modulating AMPK and suppressing the expression of *sirtuin-1 (SIRT1)*, *Beclin-1*, and *BNIP3*, thereby enhancing cell survival [90]. In a recent study, berberine activated the SIRT6—AMPK—FOXO3a axis, promoting mitochondrial biogenesis and PINK1–Parkin–mediated mitophagy, which protects cardiomyocytes during chronic intermittent hypoxia [91]. It also exerts anti-inflammatory effects by inhibiting NF-κB and PI3K/Akt signaling pathways, thereby reducing inflammatory mediators and myocardial apoptosis in I/R injury [92]. In H9c2 cells subjected to H/R, berberine enhances autophagic flux, preserves mitochondrial membrane potential, and lowers ROS and MDA levels, demonstrating a clear antioxidant defence [93].

Tetrahydropalmatine (THP), a principal alkaloid of *Corydalis yanhusuo*, has demonstrated cardioprotective effects, particularly under H/R stress [94]. In H9c2 cardiomyocytes subjected to H/R injury, THP significantly reduced intracellular and mitochondrial ROS levels, preserved mitochondrial membrane potential (ΔΨm), elevated cellular ATP content, decreased LDH release, and increased cell viability, highlighting its antioxidative and anti-apoptotic properties. THP suppressed excessive mitophagy by inhibiting phosphorylation of Unc-51-like kinase 1 (ULK1) at Ser555 and FUN14 domain-containing 1 (FUNDC1) at Ser17, key regulators of autophagy initiation, thereby reducing caspase-3 activation and apoptotic cell death [95]. In vivo study, THP treatment in a rat model of acute myocardial infarction led to improved cardiac function (higher ejection fraction); reduced infarct size; decreased oxidative stress markers (MDA) and pro-apoptotic proteins (*Bax and caspase-3*), alongside increased antioxidant enzyme activities (SOD, CAT, and GSH-Px); and enhanced phosphorylation of the PI3K/Akt survival pathway [96].

### 4.3. Saponins

Saponins, amphiphilic glycosides derived from medicinal plants, exhibit decisive anti-hypoxic and cardioprotective actions through their antioxidative, anti-inflammatory, mitochondrial-stabilizing, and signaling pathway-modulating properties [97].

Mechanism of Action:

They can activate antioxidant pathways, such as the Nrf2/HO1 pathway, and inhibit pro-inflammatory pathways, including NF-κB and the p38 MAPK/JNK pathway. Some Saponins promote angiogenesis and improve endothelial function by modulating HIF1α and VEGF expression.

Experimental Evidence:

Ginsenoside Rg1 (*Panax ginseng*) has been shown to significantly reduce myocardial infarct size, improve cardiac function, and suppress inflammation in rat models of myocardial I/R injury [98]. In vivo, Rg1 attenuates cardiac inflammation by inhibiting macrophage polarization and suppressing Absent in Melanoma 2 (AIM2) inflammasome activation, resulting in lower levels of CK-MB and LDH, as well as reduced fibrosis [99]. In vitro, Rg1 protects H9c2 cardiomyocytes from H/R injury by activating the Nrf2/HO-1 antioxidant pathway, decreasing ROS, maintaining mitochondrial membrane potential, and inhibiting JNK-mediated apoptosis [100].

Dioscin, a steroidal saponin, offers potent mitochondrial protection. In mouse models of MI/R injury, dioscin markedly reduces ROS generation and restores activities of SOD, CAT, GPx, and glutathione (GSH), resulting in improved cardiac function [101]. In cardiac H9c2 cells, dioscin prevents mitochondrial apoptosis (evidenced by reduced cytochrome c release, *Bax/Bcl-2* ratio normalization, and preserved ΔΨm) and decreases oxidative stress via SOD upregulation. Moreover, in MI animal models of acute myocardial infarction, dioscin preserves the mitochondrial Krebs cycle and respiratory enzyme activities, reduces ROS, and rescues cardiac dysfunction [101]. Diosgenin, another steroidal saponin, also exerts cardioprotective effects. In rat myocardial I/R inflammation models, diosgenin reduced infarct size, improved left ventricle function, and decreased pro-inflammatory cytokines (*TNF-α and IL-1β*) and myocardial myeloperoxidase (MPO) activity by inhibiting NF-κB signaling and p38-MAPK/JNK pathways [102].

Astragaloside IV, a saponin from *Astragalus membranaceus*, promotes angiogenesis and improves endothelial function by modulating *HIF-1α* and *VEGF* expression under hypoxic conditions [103]. Astragaloside IV (AS-IV), a principal saponin from *Astragalus membranaceus*, has demonstrated potent anti-hypoxic and cardiovascular protective effects. In vitro, AS-IV facilitates angiogenesis and endothelial recovery under hypoxic stress by enhancing the stabilization of *HIF-1α* and promoting *VEGF* expression. Specifically, in human umbilical vein endothelial cells (HUVECs) subjected to hypoxia, AS-IV activated the PI3K/Akt signaling pathway, resulting in increased HIF-1α accumulation and enhanced *VEGF* transcription, which restored tube formation and cellular migratory function in chick chorioallantoic membrane assays [104]. Furthermore, AS-IV-induced SUMOylation has been shown to stabilize HIF-1α levels in endothelial cells, thereby sustaining *VEGF* signaling and enhancing angiogenesis despite prolonged hypoxic conditions [105].

In myocardial I/R injury models in rats, post-ischemic administration of AS-IV improved function, reduced infarct size, and decreased apoptotic cell death through the upregulation of *HIF-1α* and its downstream target iNOS; these protective effects were reversed by the pharmacologic or genetic inhibition of *HIF-1α* [106]. Additionally, AS-IV produced anti-inflammatory and antioxidant effects, including the inhibition of the TLR4/NF-κB axis and a reduction in myocardial pro-inflammatory cytokines and apoptotic markers (*Bax and caspase-3*), thereby mitigating tissue injury [107]. It also modulated metabolic stress by activating the Nrf2/HO-1 pathway and normalizing succinate and lysophospholipid metabolism, resulting in reduced ROS accumulation [101].

Saponins present in common foods are generally regarded as safe at standard dietary intake levels; however, purified or high-dose saponin extracts may exhibit toxic or hemolytic effects. If intake exceeds availability, mild gastrointestinal irritation and reduced food intake may be observed. If the doses are at high concentrations, liver toxicity and nephrotoxicity have been reported in animal models. With respect to safer doses, subacute studies showed that repeated oral doses below 250 mg/kg/day were a tolerable dose, while exceeding 500 mg/kg/day may increase liver stress markers, even though traditional cooking and fermentation are the ways to reduce saponin content in several foods like legumes and quinoa so that the toxicity effect can be mitigated. EFSA or FDA, like food safety authorities, allow saponins and purified extracts in limited quantities. All these acceptances still do not support the safety of saponins [108,109,110].

### 4.4. Polysaccharides

Polysaccharides extracted from medicinal plants and fungi show immunomodulatory and antioxidant effects that support cardiovascular health. Among them, astragalus polysaccharides, in HCMECs treated with sodium dithionite (simulating hypoxia/reoxygenation), showed that the astragalus polysaccharide (APS) at 50–100 μg/mL significantly increased cell viability and reduced apoptosis (*p* < 0.01). It also lowered intracellular ROS and Ca^2+^ levels in a dose-dependent manner [111].

Experimental Evidence:

Ganoderma polysaccharides, derived from *Ganoderma lucidum*, mitigate oxidative stress and inhibit the production of inflammatory cytokines in myocardial hypoxia models, thereby promoting cardiac tissue regeneration. The Ganoderma atrum polysaccharide (PSG-1) protects neonatal rat cardiomyocytes from anoxia/reoxygenation injury by increasing cell viability and reducing LDH and MDA levels. It enhances the activity of antioxidant enzymes (MnSOD, CAT, and GPx) and decreases ROS and apoptosis. PSG-1 preserves mitochondrial membrane potential and inhibits the release of cytochrome c. It also downregulates the activation of caspase-9 and -3 and modulates Bcl-2 family proteins. These effects highlight its antioxidative and mitochondrial-protective role in hypoxic cardiac injury [112,113].

## 5. Application of Natural Compounds with Antioxidant and Anti-Inflammatory Property in Cardiovascular Disease

**Figure 2:**
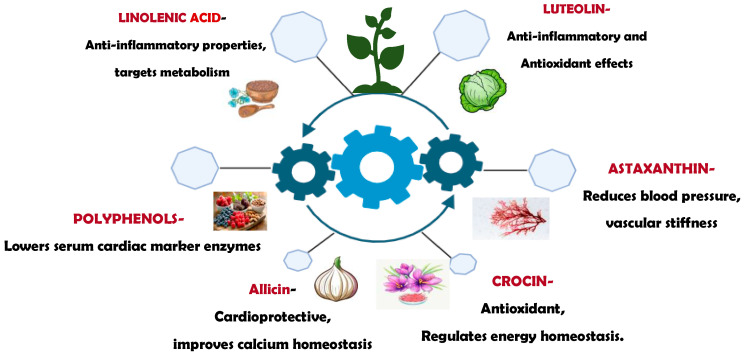
Various natural compounds can be potential therapeutics in CVDs.

Natural compounds are a promising approach for treating oxidative stress and inflammation in cardiovascular diseases. Such active components can be isolated from various medicinal plants [114,115,116]. Natural compounds derived from medicinal plants exhibit lower toxicity and offer a favorable safety profile. Compounds with therapeutic potential, supported by evidence, are listed in Table 1, Table 2, Table 3 and Table 4. Therefore, with these advantages, natural antioxidants and anti-inflammatory compounds can be utilized to target oxidative and inflammatory stress in the clinical treatment of CVD in the near future. Here, we can see how different plant-based medicinal compounds can act as therapeutics in CVD, as shown in Figure 2.

The cardioprotective effects of alkaloids have been attributed to their anti-inflammatory, antiapoptotic, and antioxidant activities, often acting via pathways, such as p38 MAPK signaling. For example, alkaloids from *Corydalis herdersonii*, which are rich in isoquinoline alkaloids, have been shown to improve cardiac function and reduce cell death after experimentally induced myocardial infarction in mice [159].

Myocardial injury, cardiac structure improvement, enhancement of antioxidant enzyme levels, and attenuation of inflammatory and apoptotic-like conditions are reduced by treatment with terpenoids. Monoterpenes, such as thymol, hinokitiol, and carvacrol, as well as diterpenes like ferruginol, have shown promising cardioprotective effects in animal models. Thymol has been shown to improve cardiac injury markers and heart function in rats by reducing inflammation and oxidative stress [160].

Plant-derived or food-based polyphenols have been reported to have a continuous intake in human clinical trials, resulting in significant reductions in cardiovascular events and improvements in surrogate markers. In a large, multicenter, randomized clinical trial involving nearly 7447 samples observed for 5 years, a 37% relative reduction in death rates among patients with CVD taking polyphenol-rich feeds was found [161].

### 5.1. Phytosterols

Phytosterols are plant-derived compounds that are similar to cholesterols in structure and are known to have a cholesterol-lowering effect in humans. A subcellular antioxidant mechanism, mediated through the modulation of cell signaling, is another mechanism exhibited by β-Sitosterol, which has shown protection with respect to H9c2 cells against hypoxia/reoxygenation-induced apoptosis [162].

#### Sitostenol

Sitostenol showed protection against the accumulation of cholesterol-rich plaques along arterial walls, which finally results in atherosclerosis. In this way, it will preserve cardiac health [163]. The prolonged activation of HIF1α leads to an increase in the level of inflammatory cytokine synthesis, which can be inhibited by fucosterol and thereby alleviates inflammation [164].

### 5.2. Polyphenols

Polyphenolic-rich extracts lowered the serum cardiac marker enzymes like LDH, aspartate transaminase (AST), and alanine transaminase (ALT), in turn reducing myocardial cell necrosis, which can be the result of an increase in lipid peroxidation [25].

#### Luteolin

The luteolin is a polyphenol, specifically a flavonoid polyphenol, which is a naturally occurring compound found in carrots, parsley, and peppers. It has both antioxidant and anti-inflammatory effects. Luteolin, along with apigenin, inhibits lipopolysaccharide-induced nitrite production in a dose-dependent manner due to suppression of inducible NO synthase. COX-2-mediated reactions and 15-LOX activities are also inhibited by luteolin [118].

### 5.3. Fatty Acid

#### Linolenic Acid

The anti-inflammatory property of linolenic acid extracted from *Ocimum sanctum* has been demonstrated to inhibit specific inflammatory molecules, such as the cyclooxygenase and lipoxygenase pathways of arachidonate metabolism [165].

### 5.4. Carotenoids

#### 5.4.1. Astaxanthin

As a carotenoid of the xanthophyll group, astaxanthin has potent antioxidants and anti-inflammatory properties due to its molecular structure, which favors the neutralization of reactive oxygen and nitrogen species. A study using spontaneously hypertensive rats (SHRs) found that astaxanthin supplementation significantly reduced systolic pressure and induced significant histological changes in the aorta, associated with decreased vascular stiffness and blood pressure [166,167]. Astaxanthin at μa 10 uM concentration significantly reduces the expression of pro-inflammatory markers, such as *IL-β*, *IL-6*, *TNF-α*, *iNOS*, and *COX-2* [167].

#### 5.4.2. Crocin

Crocin is a water-soluble carotenoid. It was isolated from *Crocus sativus* [saffron] and *Gardenia jasminoides* [168]. Crocin exhibited higher radical scavenging activity and antioxidant potential compared to gallic acid, ascorbic acid, and kaempferol [169]. In diabetes, crocin has shown cardio-protective mechanisms when tested in both in vivo and in vitro models. Crocin increases the phosphorylation of AMPK, which is a master regulator of cellular energy homeostasis in cardiac muscles [170]. When isolated cardiomyocytes are exposed to a high glucose concentration, crocin exhibits a protective role against the apoptosis pathway [171,172].

### 5.5. Organosulfur Compounds

#### Allicin

Allicin is a sulfur-containing compound in garlic. It possesses cardio-protective properties, including the reduction in blood pressure, regulation of blood lipids, prevention of atherosclerosis, and protection against myocardial injury [173]. The molecular mechanism of allicin was elucidated, as it significantly reduced serum levels of *IL-1β*, *IL-6*, and *TNF-α.* Additionally, calcium homeostasis improved in cardiomyocytes. Furthermore, allicin downregulated calcium transportation-related calcium/calmodulin-dependent protein kinase II (CaMKII), which was observed in both smooth muscle cells and cardiomyocytes [174]. It can also enhance the activity of SOD, CAT, and GSH-Px, thereby improving systolic and diastolic function in the myocardium of rats experiencing MIR injury [175].

## 6. Application of the Role of Nutritional Compounds in Diet Against Cardiovascular Diseases

A well-balanced diet, including essential macronutrients such as carbohydrates, proteins, and fats, as well as micronutrients like vitamins and minerals, provides the proper energy needed for our physical and physiological activities, including blood circulation, breathing, sleeping, and day-to-day life activities. A deficiency of these essential nutrients can cause undernutrition and other complications, including fatigue, weakness, and shortness of breath.

Berries, suchs blueberries, blackberries, and cranberries, contain polyphenols and anthocyanins, which are antioxidants that help combat hypertension, hyperlipidemia, and anemia [176]. Citrus fruits like lemon and orange, which have flavonoids, limonoids, alkaloids, and carotenoids, showed anti-cholinesterase activity, xanthine oxide modulators, and iron absorption. These, together with cumulative effects, help produce more oxygen [176]. Green, leafy vegetables contain a vast array of polyphenols, tannins, beta-carotenes, and vitamin K, which can have direct effects on microbial metabolism by inhibiting oxidative phosphorylation. Thus, they have potential towards hypoxic damage [176].

Docosahexaenoic acid (DHA) is essential for optimal fetal development and a healthy cardiovascular system [177]. DHA in the hypoxic pulmonary hypertension rat model reduces the pulmonary arterial systolic pressure and improves right ventricular hypertrophy. It also prevented proliferation, migration, and phenotype switching of pulmonary arterial smooth muscle cells induced by hypoxia [178,179].

Eicosapentaenoic acid [EPA] is also a long-chain n-3 PUFA, which has been proven to have anti-inflammatory, anti-hypertensive, and antithrombotic properties and even lowers triglycerides [180,181,182]. The EPA reportedly reduced systolic pulmonary arterial pressure, providing anti-inflammatory effects by enhancing the expression of pulmonary GPR120 mRNA in an MCT-induced PAH rat model [182].

Apples, grapes, berries, tomatoes, onions, lettuce, etc., contain flavanols such as quercetin, kaempferol, and myricetin, which have been found to exhibit therapeutic benefits in CVDs, including endothelial dysfunction, coronary artery disease, cardiac fibrosis, and myocardial infarction [183,184,185]. Onions, broccoli, and tea contain isorhamnetin, kaempferol, and myricetin, which regulate systolic blood pressure and glycemic levels. Catechins, including epicatechins, present in apricots, cocoa, and red grapes, have been shown to reduce mean arterial pressure. Anthocyanidins like cyanidin, delphinidin, malvidin, peonidin, etc., have the potential to reduce the risk of myocardial infarctions, which are present in red cabbage, cherries, and colored fruits [186,187].

## 7. Multitherapeutic Approach for Addressing CVDs

The application of individual antioxidant and anti-inflammatory compounds to treat CVDs has not been achieved with great success. A new approach needs to be explored. In this path, a combination of compounds may be an effective way to achieve better outcomes. The combination of antioxidants has been widely studied. Vitamin E with crocin combination was administered on a model of the isolated heart of a rat; as a result, a reduction in infarct size was marked, which was not similar when antioxidants were administered individually. Also, a combination of vitamin C and vitamin E was promising in the treatment of patients suffering from acute myocardial ischemia [AMI]; even when both are combined with vitamin A, the results were promising [188,189,190,191].

Trimetazidine, when combined with berberine, exhibited a promising therapeutic effect on endothelial function in patients with congenital heart disease [CHD]. This combination increased blood NO content, promoted the endothelium-dependent relaxation function of the brachial artery, and was involved in a controlled clinical protocol (CCP) [192].

The cardiovascular benefits of ginseng and its constituents have been assessed through several multicenter randomized clinical trials. An Asian ginseng extract, administered at 3 g daily for 12 weeks to patients with hypertension and diabetes, demonstrated a reduction in arterial stiffness and systolic blood pressure compared to a placebo in the study [193].

A plant-based diet was associated with a significantly lower risk of both cardiovascular mortality and CVD incidence, which involved 410,000 participants. This study provides substantial evidence to suggest a possible protective role of plant-based dietary patterns, although not all plant foods are equally beneficial. Exploring healthy plant-based diets can be a potential way to address pre-existing CVDs [194].

A recent multicenter trial has evaluated the efficacy and safety of Chinese herbal compounds, such as Qing-Xin-Jie-yu granule, in treating ischemic cardiovascular lesions, with outcome measures including myocardial ischemia and plaque stabilization among large, diverse patient cohorts [195].

Such a new combinational formula can offer a promising future perspective for therapeutics, and through this approach, cardiovascular disease treatment may be improved with a better success rate.

## 8. Future Perspective

The future of natural compounds with antioxidant and anti-inflammatory properties in cardiovascular treatment is auspicious. Advances in mechanistic understanding, technology-driven discovery, and the move toward personalized medicine are paving the way for these bioactive compounds to become integral components of cardiovascular care. There is a growing recognition of the potential for natural anti-inflammatory and antioxidant molecules to play a significant role in the prevention and treatment of CVD. As awareness of the limitations and side effects of synthetic drugs increases, attention is shifting toward bioactive compounds derived from plants, fruits, vegetables, herbs, and other natural sources. Flavonoids, such as quercetin, catechins, and rutin, found in fruits, vegetables, tea, and cocoa, have shown potent antioxidant and anti-inflammatory effects. They modulate Nrf2/ARE signaling and have favorable effects on vascular health, blood pressure, lipid profiles, and inflammation [117,196]. Colchicine-like molecules have been utilized in drug repurposing due to their demonstrated strong anti-inflammatory properties, which have now been validated in clinical trials for the secondary prevention of cardiovascular events [197,198]. Recent research on nano-encapsulated flavonoids has shown enhanced delivery, increased bioavailability, and greater efficacy in targeting cardiovascular oxidative stress and inflammation [199]. Ginsenosides derived from ginseng modulate oxidative stress, reduce apoptosis, and protect cardiac tissue. Combining natural antioxidants and anti-inflammatories with current therapies for CVD may enhance efficacy and minimize side effects [200]. Advances in pharmacogenomics and metabolomics may allow for the tailored use of natural compounds based on individual patient profiles. Ongoing research will focus on resolving clinical uncertainties, optimizing delivery systems, and integrating these bioactives into evidence-based cardiovascular care.

The problem of low bioavailability of natural compounds can be overcome by the application of new technological adaptations, such as nano-encapsulated polyphenols in food-grade polymers and lipids, which result in improved oral absorption, enhanced intestinal uptake, and greater systemic availability for cardiovascular protection [201]. Liposomal formulations of antioxidants, such as vitamin E, tocotrienols, and N-acetylcysteine, enhance their stability, cellular uptake, and targeted delivery to cardiac and vascular tissues, resulting in improved therapeutic efficacy in animal models and preliminary human trials [202]. Food-grade biopolymer nanoparticles and PEGylated nanocarriers offer sustained release, mucoadhesive properties, and enhanced bioavailability for natural products in the prevention of cardiovascular disease [194].

While challenges such as standardization, regulatory approval, and large-scale clinical validation remain, ongoing research and innovation are expected to overcome these barriers. Ultimately, natural compounds—either alone or as adjuncts to conventional therapies—hold significant potential to improve cardiovascular outcomes, reduce disease burden, and enhance patient quality of life in the years to come [191].

## 9. Conclusions

Oxidative and inflammatory processes are closely related to several metabolic diseases and ageing-related degenerative disorders in the body. These stresses are considered critical factors in the most extensive burdens in the world, such as cancer and cardiovascular diseases. The therapeutic efficacy of natural drugs and their active ingredients, which have the potential to combat oxidative and inflammatory damage, has received considerable attention. Several research activities have shown that the individual or combination of small numbers of antioxidants and anti-inflammatory compounds in the form of diet has a protective role against CVD. Links among oxidative stress, pro-inflammatory systemic environment, and CVD are well established through various scientific evidence. Therefore, one approach to mitigate stress without excessive toxicity to the body is the use of natural compounds. For more than three decades, it has been established that supplementation with antioxidants and anti-inflammatory molecules has beneficial therapeutic effects. To support this, a meta-analysis of randomized controlled trials involving nearly a thousand subjects was conducted, and it found that antioxidant supplementation does not lead to an increased risk of cancer. Due to the incorrect selection of compounds, inappropriate dose, improper combination, or incorrect duration, natural compounds may not exert the expected therapeutic effects. Like personalized medicine, individual antioxidant and anti-inflammatory deficiencies must be diagnosed, and then, therapy should begin to achieve the expected results. Dietary intake holds low doses of such potential compounds. However, when combined with two or three antioxidants and anti-inflammatory molecules, it could be safe and effective. To summarize, therapy with natural compounds is not ineffective; it is necessary to identify optimal approaches through research on such compounds available in nature and to enhance their effects in diet and nutrition. Through such evidence, new curative and preventive therapies can be developed to serve humankind.

## Figures and Tables

**Table 1:** Natural antioxidants and anti-inflammatory compounds.

Category	Compounds	Activity
**Polyphenols**	Resveratrol	Ameliorate OS by increasing Nrf2 expression Also decrease inflammation through TLR4/NF-kB signaling pathway [117] Inhibits ferroptosis via inducing KAT5/GPX4 in MI [76]
	Quercetin	Scavenging and inhibition of ROS and induction of Nrf2/HO-1 expression [117] Downregulates the expression of adhesion molecules [77]
	Curcumin	Decrease myocardial apoptosis by activating JAK2/STAT3 pathway, thus reducing OS-damage [117]
**Carotenoids**	Lycopene	Inhibit mPTP opening via modulation of *Bax* and *Bcl-2* [118]
	Crocin	In the heart, there is a regulation of SIRT1/Nrf2 signaling and related endoplasmic reticulum stress. In the brain, a reduction in *HIF1α* and caspase-3 was seen [67,118]
	Beta-carotene	Inhibit NF-kB pathway [119]
	Astaxanthin	Activate Nrf2/HO-1 pathway, regulate the miR-138/HIF1α axis. SOD1 and 2 expression will be enhanced [119]
	Lutein	Decrease SOD, CAT, and GTX activity [120]
**Vitamins**	All-trans retinoic acid	Downregulation of MAPK signaling [121]
	Vitamin C	Decrease SOD activity and PI3k-Akt signaling pathway [122]
	Vitamin D	Reduce inflammation by RhoA/ROCK/NF-kB pathway; activate Nrf2/HO-1 pathway [123]
	Vitamin E	Downregulation of GPx (1, 5 and 6) and MPO [124]
	Folic acid	Inhibition of NMDAR [125]

**Table 2:** Natural compounds from diverse origins with antioxidant and anti-inflammatory Properties.

Category	Compound	Activity
**Plant-derived natural products**	Epigallocatechin gallate—catechin group of flavanoid	Anti-inflammatory, anti-cancer, anti-obesity, induces autophagy and promotes synaptic plasticity, reduces neuroinflammation [126]
	Capsaicin—alkaloid	Vasodilator, antibacterial anticancer, ROS disruption of mitochondrial membrane transition potential [35,126]
	Ferulic acid—polyphenol	Anti-oxidant; regulates the activities of antioxidant and lipogenic enzymes [127]
**Animal-derived natural products**	Venom	Anti-inflammatory [127]
	Cantharidin—terpenoid	Anti-inflammatory [128]
	Bufalin—cardiotonic steroid	Anti-inflammatory [129]
	Tetrodotoxin—non-proteinaceous neurotoxin	Anesthetic and analgesic [130]
	Ursodeoxycholic acid—secondary bile acid	Antitumor [130]
**Microbe-derived NPs**	Lovastatin—class of polyketide	Lowering blood lipids and cholesterol [130]
	Ganospirones B—tetracycline terpenoid	Anti-inflammatory and anti-renal fibrosis; promotion of neural stem cell proliferation; inhibition of JAK3 kinase [131]
	Applanatumin A—meroterpenoid dimer	Antifibrotic [132]
	Sinensilactam A—a hybrid metabolite	Antifibrotic Smad3 phosphorylation inhibitor [133]

**Table 3:** Different secondary metabolites with antioxidant and anti-inflammatory properties.

Category	Compounds	Activity
**Phenolic acids**	Gingerol	Antioxidant activity [134]
	Caffeic acid	Antioxidant activity [134]
	Paeonol	Upregulates *bcl-2* levels and suppresses apoptosis [135,136]
**Flavonoids**	Apigenin	Attenuates cardiac impairment by inhibiting TGF-β_1_-mediated SMAD pathway [137]
**Glycosides**	Ginsenoside Rb1	Promotes mitophagy via AMPKA phosphorylation [138]
	Ginsenoside Rb2	Inhibits p300-mediated SF3A2 acetylation at lysine10 [139]
	Geniposide	Suppresses NLRP3 inflammasome, AMPK signalling pathway [140,141]
	Catalpol	Attenuates cardiac dysfunction: regulates the apelin/APJ pathway [142]
	Hydroxysafflor yellow A	Anti-oxidant, anti-inflammatory, and neuroprotective effect [143]
**Terpenoids**	Bitulin	Regulates the Siti1/NLRP3/NF-kB singling pathway [144]
	Artemisinin	Inhibits NLRP3 inflammasome [145]
	Oridonin	Inhibits NLRP3 inflammasome [34]
**Alkaloids**	Berberine	Inhibits macrophage Wnt5a/β-catenin pathway [146]
	Colchicine	Anti-inflammatory activity [147]
**Quinone**	Dihydrotanshinone-1	Protects cardiomyocytes via PKM2 glutathionylation [148]
	Salvianolic acid	Upregulates the Nrf2 signalling pathway [149]

**Table 4:** Active plant-derived ingredients and their mode of action against cardiovascular disease.

Active Ingredient	Model	Mechanism of Action
**Icarin**	High-glucose and adenovirus-induced cardio myopathy in neonatal C57 mice	Increases Apelin/SIRT3 [150]
**Hesperidin- Flavonoid**	Nitric oxide deficiency-induced cardiovascular remodeling	Regulates Nrf2/ARE/HO-1 and TGF-beta/Smad3 signal transduction. Ischemia–reperfusion [138]
**Rutin**	Cobalt chloride-induced hypoxic injury in H9c2cells	Modulation of Akt, p-Akt, p38, and p-p38; of HIF-1, BAX, and caspase [150]
**Astragalin**	Myocardial ischemia/reperfusion(I/R) injury in isolated rat heart	Inducing autophagy; increasing the expression of nrf2/HO-1/NADPH/NQO1 heart failure, reduces ROS; inflammation; myocardial apoptosis; enhances Bcl-2 [151]
**Hyperoside**	High-glucose-induced oxidative stress in cardiac cells	Enhances p-AKT/AKT and p-Nrf2/Nrf2; reduces myocardial apoptosis and levels of ROS [152]
**Naringenin**	H_2_O_2_-induced oxidative stress in cardiomyocytes	Increases the activity of antioxidant enzyme; increases Nrf2 signaling pathway [153]
**Delphinidin**	Myocardial ischemia/reperfusion injury in rats	Reduces expression of STAT1 [154]
**Daidzein**	Isoproterenol-induced apoptosis in H9c2 cardiomyoblast	Increases Akt activation [155]
**Petunidin**	Myocardial ischemia/reperfusion injury in rats	Increases *Bcl-2* expression; reduces *NOX4* and *Bax* expression [156]
**Mangiferin**	Myocardial ischemia/reperfusion injury in rats	Reduces phosphorylation of p38 and JNK; phosphorylation of ERK1/2 [157]
**Malvidin**	Isoproterenol-induced apoptosis in H9c2 cardio myoblast	Increases Nrf2/HO-1 signaling pathway; reduces NF-kB signaling [158]

## Data Availability

No new data were created or analyzed in this study. Data sharing is not applicable to this article.
